# Effects of PDE5 Inhibitors and sGC Stimulators in a Rat Model of Artificial Ureteral Calculosis

**DOI:** 10.1371/journal.pone.0141477

**Published:** 2015-10-28

**Authors:** Peter Sandner, Hanna Tinel, Giannapia Affaitati, Raffaele Costantini, Maria Adele Giamberardino

**Affiliations:** 1 Bayer Health Care AG – Global Drug Discovery, Department of Cardiology – Pharma Research Center Wuppertal, Wuppertal, Germany; 2 Institute of Pharmacology, Hannover Medical School, Hannover, Germany; 3 Pathophysiology of Pain Laboratory, Ce.S.I., “G. D’Annunzio” University of Chieti, Chieti, Italy; 4 Department of Medicine and Science of Aging, “G. D’Annunzio” University of Chieti, Chieti, Italy; 5 Institute of Surgical Pathology, “G. D’Annunzio” University of Chieti, Chieti, Italy; Maastricht University, NETHERLANDS

## Abstract

Urinary colics from calculosis are frequent and intense forms of pain whose current pharmacological treatment remains unsatisfactory. New and more effective drugs are needed to control symptoms and improve stone expulsion. Recent evidence suggested that the Nitric Oxide (NO) / cyclic guanosine monophosphate (cGMP) / phosphodiesterase type 5 (PDE5) system may contribute to ureteral motility influencing stone expulsion. We investigated if PDE5 inhibitors and sGC stimulators influence ureteral contractility, pain behaviour and stone expulsion in a rat model of ureteral calculosis. We investigated: a)the sex-specific PDE5 distribution in the rat ureter; b)the functional *in vitro* effects of vardenafil and sildenafil (PDE5 inhibitors) and BAY41-2272 (sGC stimulator) on induced ureteral contractility in rats and c)the *in vivo* effectiveness of vardenafil and BAY41-2272, alone and combined with ketoprofen, vs hyoscine-N-butylbromide alone or combined with ketoprofen, on behavioural pain indicators and stone expulsion in rats with artificial calculosis in one ureter. PDE5 was abundantly expressed in male and female rats’ ureter. *In vitro*, both vardenafil and BAY41-2272 significantly relaxed pre-contracted ureteral strips. *In vivo*, all compounds significantly reduced number and global duration of “ureteral crises” and post-stone lumbar muscle hyperalgesia in calculosis rats. The highest level of reduction of the pain behaviour was observed with BAY41-2272 among all spasmolytics administered alone, and with the combination of ketoprofen with BAY41-2272. The percentage of stone expulsion was maximal in the ketoprofen+BAY41-2272 group. The NO/cGMP/PDE5 pathway is involved in the regulation of ureteral contractility and pain behaviour in urinary calculosis. PDE5 inhibitors and sGC stimulators could become a potent new option for treatment of urinary colic pain.

## Introduction

In the general population, 12 to 15% of subjects develop kidney stones throughout their life span, with incidence rates that have steadily increased in recent years [1–4]. Acute ureteral obstruction and urinary colics from calculosis are therefore quite frequent. In addition, the management of renal and ureteral stones by extracorporeal shock wave lithotripsy (ESWL) and the subsequent expulsion of stone fragments are most often accompanied by colic pain [5]. These urinary colics are among the most frequent and intense forms of pain that a human being can experience. In addition to spontaneous pain, patients undergoing colics also develop marked muscular hyperalgesia in the area where the pain is perceived, which long outlasts the painful episodes [6,7]. Treatment of pain symptoms from urinary calculosis with the available pharmacological compounds (Non-Steroidal Antiinflammatory Drugs-NSAIDs /classic spasmolytics / central analgesics) is very often unsatisfactory and there is an imperative need for new, more effective drugs able to fully control the symptomatology but also actively improve stone expulsion [8,9]. Therefore, the understanding of the specific physiology of the ureteral muscle opens a new avenue of pharmacological treatment. Previous studies have shown that a combination treatment of NSAIDs with compounds acting on ureteral contraction, such as calcium channel blockers, Nitric Oxide-donors, inhibitors of phosphodiesterases and blockers of alpha1 adrenergic receptors, has some efficacy in colic pain from urinary calculosis [10–12]. Although the effects of alpha blockers are broadly investigated in preclinical and clinical studies, less is known about the impact of the Nitric Oxide (NO) / cyclic guanosine monophosphate (cGMP) / phosphodiesterase type 5 (PDE5) system on ureteral contractility, pain behaviour and stone expulsion. The NO/cGMP/PDE5 pathway can be pharmacologically influenced at the level of cGMP production by NO-donors or direct stimulation of the soluble guanylate cyclase (sGC) and at the level of cGMP degradation by inhibition of the PDE5. It is well accepted that phosphodiesterase type 5 inhibitors work on the lower urinary tract and PDE5 inhibitors are used as first line therapy for erectile dysfunction, but more recently the PDE5 inhibitors, namely sildenafil, vardenafil and tadalafil, were found to be effective for the treatment of benign prostatic syndrome (BPS), symptomatic benign prostatic hyperplasia (BPH) and lower urinary tract symptoms (LUTS) with the recent approval of tadalafil in these indications [13–16]. The mechanism of action is at least in part based on relaxation of smooth muscle cells in penile tissues but also prostatic, bladder and ureteral muscle [17,18]. It has also been shown that PDEs are expressed in the rat, pig and human ureteral tissues and that PDE inhibitors could also influence ureteral contractility in vitro in organ bath experiments [19,20]. However, due to the mode of action, the efficacy of PDE5 inhibitors is limited in conditions with low endogenous NO/cGMP supply, since they block cGMP degradation only. In contrast, the so-called sGC stimulators, which are a new class of compounds, can directly stimulate the cytosolic soluble guanylate cyclase (sGC) independently of endogenous NO levels [21]. Since these compounds are less NO-dependent, this may result in a higher efficacy compared to the PDE5 inhibitors. The effects of these compounds on the lower urinary tract are almost unknown and the role of this system for ureteral contractility is not yet clear. There is scarce *in vitro* evidence for the effects of PDE5 inhibitors and a complete lack of *in vivo* studies on the impact of sGC stimulators on ureteral function, especially for stone expulsion. In addition, so far nobody has focused on a direct comparison of PDE5 inhibitors with sGC stimulators for the treatment of ureteral colics. Most important, there is to our knowledge no study describing the overall symptomatic pain behaviour *in vivo* for these classes of compounds. In this study we therefore used a rat model of artificial ureteral calculosis set up by our group to test the effects of PDE5 inhibitors and sGC stimulators on colic pain and referred muscle hyperalgesia vs placebo and vs a classic spasmolytic (hyoscine-N-butylbromide)[22]. This model is particularly suitable for testing active compounds on urinary pain. Rats with an artificial stone in one ureter exhibit pain behaviours that mimic both the painful colic episodes and the referred muscle hyperalgesia experienced by humans [5–7,23]. The spontaneous pain behaviour, monitored through continuous video-tape recordings for several days post-stone implantation, consists of multiple “ureteral pain crises” over 4 post-operative days. Pharmacologic validation of the nociceptive nature of these episodes is provided by their reduction upon administration of major analgesics (morphine, tramadol), NSAIDs (ketoprofen, metamizol) or classic spasmolytics (hyoscine-N-butylbromide) [22,24–29]. The rats also show hyperalgesia of the obliquus externus muscle ipsilateral to the implanted ureter, revealed by a significant decrease in the vocalization threshold to electrical stimulation of the same muscle which starts on the first day after stone implantation, reaches a peak on the 2nd-3rd day, and persists for over a week. Number and global duration of ureteral crises are significantly and directly related to the extent of the muscle hyperalgesia. As already stated above, this validated animal model of artificial ureteral calculosis closely resembles the human condition of urinary colics from calculosis, as it reproduces not only the spontaneous pain perceived by patients, but also the referred muscle hyperalgesia, which is longlasting and has been shown to be correlated to the number of colics experienced [1,6,7]. As in humans, the “destiny” of the stone in implanted rats differs, as shown by autopsy findings at the end of the behavioural evaluation period; in some animals the stone appears to have been expelled, in others it has moved along the ureter, and in others it remains in the original position creating obstruction of the urinary tract. The percentage of stone expulsion can be influenced by the applied pharmacologic treatment [30]. For these reasons, in the present study we aimed at evaluating the effects of PDE5 inhibitors and sGC stimulators not only on the behaviour indicative of pain, but also on the destiny of the stone and stone expulsion rates.

In summary, the aim of the present study was to: (1) assess the phosphodiesterase 5 expression in the rat ureter, (2) directly compare the *in vitro* potency of the PDE5 inhibitors vardenafil and sildenafil and the sGC stimulator BAY 41–2272 on ureteral contractility and (3) test *in vivo* the effectiveness of the PDE5 inhibitor vardenafil and the sGC stimulator BAY 41–2272, vs the classic spasmolytic hyoscine-N-butylbromide, alone and in combination with ketoprofen (one of the most frequently employed NSAIDs for urinary colics), on behavioural indicators of urinary pain and on stone expulsion in rats with artificial ureteral calculosis. We showed here that the NO/cGMP/PDE5 pathway is involved in the regulation of ureteral contractility and pain behaviour in urinary calculosis suggesting that PDE5 inhibitors and sGC stimulators could become a potent new option for treatment of urinary colic pain either administered alone or in combination with other antinoceptive drugs.

## Materials and Methods

### Tissue sampling and PDE5 expression profiling

Male and Female Sprague Dawley rats (n = 8) with a body weight between 200–250 g were used for tissue collecting. The rats were briefly anaesthetized with a mixture of 5% Isoflurane (Baxter S.A.) in a carrier with 70% N_2_O and 30% O_2_, and consecutively euthanized by decapitation. The abdomen was opened and the ureteral tissues quickly removed, frozen in liquid nitrogen, and stored until RNA preparation. Male penile tissue (corpus cavernosum) was obtained from the male Sprague Dawley rat group. Total RNA from ureter and corpus-cavernosum tissue was isolated using RNeasy mini columns (Qiagen Inc.) and further purified by DNase digestion.

#### PDE-5 mRNA quantification

Gene expression profiling was done with a standard semiquantitative RT-PCR protocol using total RNA, as described previously [31]. In brief: the mRNA expression of PDE-5 was measured by real time quantitative PCR (TaqMan-PCR) using an ABI Prism 7700 sequence detection instrument (Applied Biosystems, Inc.). After isolation of total RNA as described above, 1 μg of total RNA was transcribed into cDNA with Superscript II RT cDNA synthesis kit (Gibco, Inc). The sequences of forward and reverse primers (designed by Primer Express 1.5 Software, Applied Biosystems, Inc.) were 5‘-ttgacggatctggagacgct-3‘ and 5‘-caccacgatggtccaaatca-3‘, respectively. The fluorogenic probe used was 5‘-6FAM-cgattgctgatggccgctttaagcc-TAMRA-3‘. During PCR amplification, 5‘ nucleolytic activity of Taq polymerase cleaves the probe separating the 5‘ reporter fluorescent dye from the 3‘ quencher dye. The threshold cycle, Ct, which correlates inversely with the target mRNA level, was measured as the cycle number at which the reporter fluorescent emission increases 10 standard deviations above background level. As housekeeping gene, beta-actin was quantified as described above, using 5′-accttcaacaccccagcca-3′ as forward primer and 5′-cagtggtacgaccagaggca-3′ as reverse primer. The PDE-5 mRNA levels, expressed as Ct, were set in relation to Ct of beta-actin which was defined as relative expression.

### Contractility experiments on isolated rat ureter

Male Wistar rats (250–300 g) were anaesthetized with ether, and the ureter was removed and placed in ice-cold calcium-free Krebs-Henseleit (KH) buffer of the following composition (in mmol/l): NaCl 112, KCl 5.9, MgCl2 1.2, NaH2PO4 1.2, NaHCO3 25, and glucose 11.5.

For measurement of isometric tension, ring segments, 2 mm in length, were mounted in a small chamber myograph. Two wires (40 μm diameter) were introduced through the lumen of the segments and mounted according to the method described for blood vessels by Mulvany and Halpern (1977)[32]. The preparations were allowed to equilibrate for 30 min in calcium-free KH solution and stretched to a length where passive tension was about 0.5 mN/mm which, in preliminary experiments, seemed to be the optimal for active force. The oscillations of the ureter were induced by a high potassium (20 mmol/l KCl) KH buffer containing 2 mmol/l CaCl_2_ followed 20 min later by a supramaximal concentration of noradrenaline (10 μmol/l). If the oscillations were sustained for 25 min, the test compound was added to the myograph chamber and the recording continued for 25 min. The oscillations before and after application of vehicle (dimethyl sulfoxide: end concentration 0.01%) or the test compound were counted and the oscillation frequency (counts/min) was calculated. The effects of vehicle or compound were expressed as a percentage of the oscillation frequency before application of vehicle or compound (which was defined as 100%).

### Behavioural Study

#### Experimental protocol

A total of 144 Female Sprague-Dawley rats (200–280 g) were used for the experiment, randomly assigned to 8 experimental groups of 18 each, for different treatments. The experimental groups were as follows:

1. Placebo [0.5% tylose, orally (p.o.), twice/day]
2. Ketoprofen (10 mg/kg/day in 0.5% tylose, p.o., divided in 2 administrations/day)
3. Hyoscine-N-Butylbromide (15 mg/kg/day in 0.5% tylose, p.o., divided in 2 administrations/day)
4. Vardenafil (PDE5 inhibitor) [3 mg/kg/day in 0.5% tylose, p.o., divided in 2 administrations/day]
5. BAY 41–2272 (sGC stimulator) [3 mg/kg/day in 0.5% tylose, p.o., divided in 2 administrations/day]
6. Ketoprofen + Hyoscine-N-Butylbromide [10mg/kg/day + 15mg/kg/day, in 0.5% tylose, p.o., divided in 2 administrations/day)
7. Ketoprofen + Vardenafil [10 mg/kg/day + 3 mg/kg/day in 0.5% tylose, p.o., divided in 2 administrations/day)
8. Ketoprofen + BAY 41–2272 [10 mg/kg/day + 3 mg/kg/day in 0.5% tylose, p.o., divided in 2 administrations/day].

The times of the 2 administrations/day were 9:30 a.m. and 7:30 p.m.

The protocol schedule was as follows:

- On day -3, all rats were operated on under general pentobarbital anaesthesia (50mg/kg, i.p.) for implantation of muscle electrodes (for subsequent testing of muscle sensitivity): bipolar wire electrodes in the obliquus externus muscle of one side (left lumbar region, L1).
- On days -2, -1 and 0, all rats underwent measurement of the vocalization threshold to electrical stimulation of the left oblique muscle, always at the same hour of day (9:00 a.m.).
- On day 0, soon after muscle measurement, all rats underwent a second operation (pentobarbital anaesthesia) for stone formation in the left ureter.
- On days +1,+2,+3,+4, they again underwent muscle vocalization threshold measurement. During this 4-day period, they were video-recorded non-stop for monitoring of ureteral pain crises.
- On day +4, all rats were suppressed via an overdose of pentobarbital and underwent autopsy for evaluation of the urinary tract condition.

In all rats, treatment started on day 0, about 5 hours after the beginning of ureteral stone implantation, and continued up to day 4.

#### Technique of muscle implantation

Under general pentobarbital anaesthesia, bipolar wire electrodes were implanted in the left obliquus externus muscle (lumbar region, L1). The isolated wires were passed under the skin towards the skull, where connectors were fixed into the parietal bones by means of small screws and dental cement, a procedure allowing electrical stimulation in the freely moving animals for vocalization threshold measurement on the subsequent days [22,26].

#### Technique of stone implantation

Under general pentobarbital anaesthesia, each rat underwent a vertical suprapubic incision; an artificial stone was formed in the upper third of the left ureter by injecting 0.02 ml of dental cement (while still fluid) in the lumen using a syringe with a 0.4 mm diameter needle, according to a technique already described in detail elsewhere [22,24, 26,27,29,33,34].

#### Assessment of spontaneous behaviour

The spontaneous pain behaviour was monitored through continuous video-tape recordings using a time-lapse system for 4 days post-stone implantation, with ultrared light for filming during the dark phase. Over the whole study period the rats were housed one per cage (plexiglass 35 x 23 x 18 cm) in a temperature / humidity-controlled environment, subjected to a 12:12 h dark-light cycle (8:00 a.m.– 8:00 p.m. artificial light), with free access to food and water [29]. The video-tape recording was interrupted for 30 min each morning (9:00–9:30) to allow sensitivity testing of the left oblique muscle and weighing of the animals from the 1^st^ day post-muscle implantation to the 4^th^ day post-stone formation, as well as administration of the experimental compounds during the treatment phase. The video-recording was also briefly interrupted at 07:30 p.m. for the evening drug administration during treatment. Tests and drug administration were delivered by an experimenter blind to treatment allocation of the animals (the product preparation was performed by a different experimenter).

On the 4^th^ day after stone implantation all rats were killed via an overdose of pentobarbital and an autopsy was performed to examine the urinary tract condition.

The spontaneous behaviour presented by stone-rats was analyzed upon reading of the video-tape recordings according to a standardized technique. The typical behaviour, as standardized and exactly described in previous papers as it is here [22,29], consists of multiple “ureteral pain crises”, i.e., a sequence of at least 3 out of 6 possible pain behaviours within a period of minimum 2 min. The six possible behaviours include: (i) hump-backed position, (ii) licking of the lower abdomen (and/or left flank), (iii) contraction of the left oblique musculature with inward moving of the ipsilateral hindlimb, iv) stretching of the body with raised abdomen, v) squashing of the lower abdomen against the floor, vi) supine position with left hindlimb adducted and compressed against the abdomen. Complexity of a crisis is evaluated on a 1–4 point arbitrary scale, based on the number of behaviours: a sequence of 3 behaviours is scored 1, sequences of 4, 5 or 6 behaviours are scored 2,3 or 4, respectively. For each rat, the video-tape was read by an experimenter blind to the kind of treatment, to count and classify the crises [22].

#### Assessment of vocalization thresholds to muscle stimulation

Vocalization thresholds were measured every day for 3 days before and 4 days after ureteral stone implantation, always at the same hour. The rat was placed in a single cage, allowed to move freely inside it, and the electrical stimuli were delivered through the connectors fixed on the skull. A constant-current stimulator was used. Stimuli consisted of 250 ms trains of 1 ms square wave impulses (frequency 200/s), delivered automatically every 2 s. Vocalization thresholds were measured according to the methods of the limits. The intensity was increased in 0.3 steps until vocalization occurred, then decreased in 0.1 mA steps until is disappeared and then re-increased at the latter rate until vocalization returned, and the corresponding values were noted in mA. The threshold was calculated as the mean of these values [26].

The use of different anaesthetic procedures in the different types of experiments performed in this study, namely, in vitro gene expression profiling, ex vivo organ bath assays and in vivo rat studies, is due to the long-term experience in all these different experimental approaches. Anaesthesia procedures were optimized to guarantee valid measurements of each parameter in these different experiments and make them comparable to previous studies.

### Statistical analysis

For the *in vitro* and organ bath experiments statistical significance was determined by paired and unpaired Student’s t-tests with P< 0.05 considered to indicate significance.

For the *in vivo* experiments, the number of 18 animals per group was determined from a statistical evaluation based on the characteristics of the behavioural parameters of the model, i.e., for a statistical power of 90% to detect a difference versus placebo and among active treatments, with P<0.01 to indicate significance.

In each rat:

- number, global duration (sum of duration of all crises) and mean complexity of crises over the 4-day post-stone-implantation period were calculated;
- muscle threshold of each post-operative day was calculated as a percentage of the pre-operative reference threshold (mean of values recorded on days -2, -1 and 0).

For each group of experimental rats, Means ± Standard Deviation (SD) were calculated of:

- number, global duration and mean complexity of crises for the 4-day post-stone intervention period;
- muscle thresholds for each evaluation day.

One-way ANOVA and ANOVA for repeated measures (in the case of thresholds) were applied to assess the effects of the various treatments on the different behavioural parameters. Post-hoc tests were applied when appropriate. The percentage of stone expulsion among groups was compared using the Fischer’s exact test.

### Ethical issues

The experimental protocol was approved by the Animal Use and Care Committee of the G. D’Annunzio University of Chieti and the Italian Health Ministry, section on Animal Experments (n.159/2008.B) and adhered to the guidelines established by the European Union for experimental procedures in laboratory animals. The study complies with the ARRIVE guidelines for research involving animals [35].

Rats were studied for the minimum period necessary to verify the effects of the drugs administered (4 days, based on previous work) and were killed at the end of the observation period via an overdose of barbiturate. It was part of the experimental design that rats presenting any sign of unnecessary suffering in the course of the observation period (such as abnormal weight loss) should be excluded from the study and sacrificed immediately [36].

## Results

### PDE5 mRNA expression

**Fig 1:**
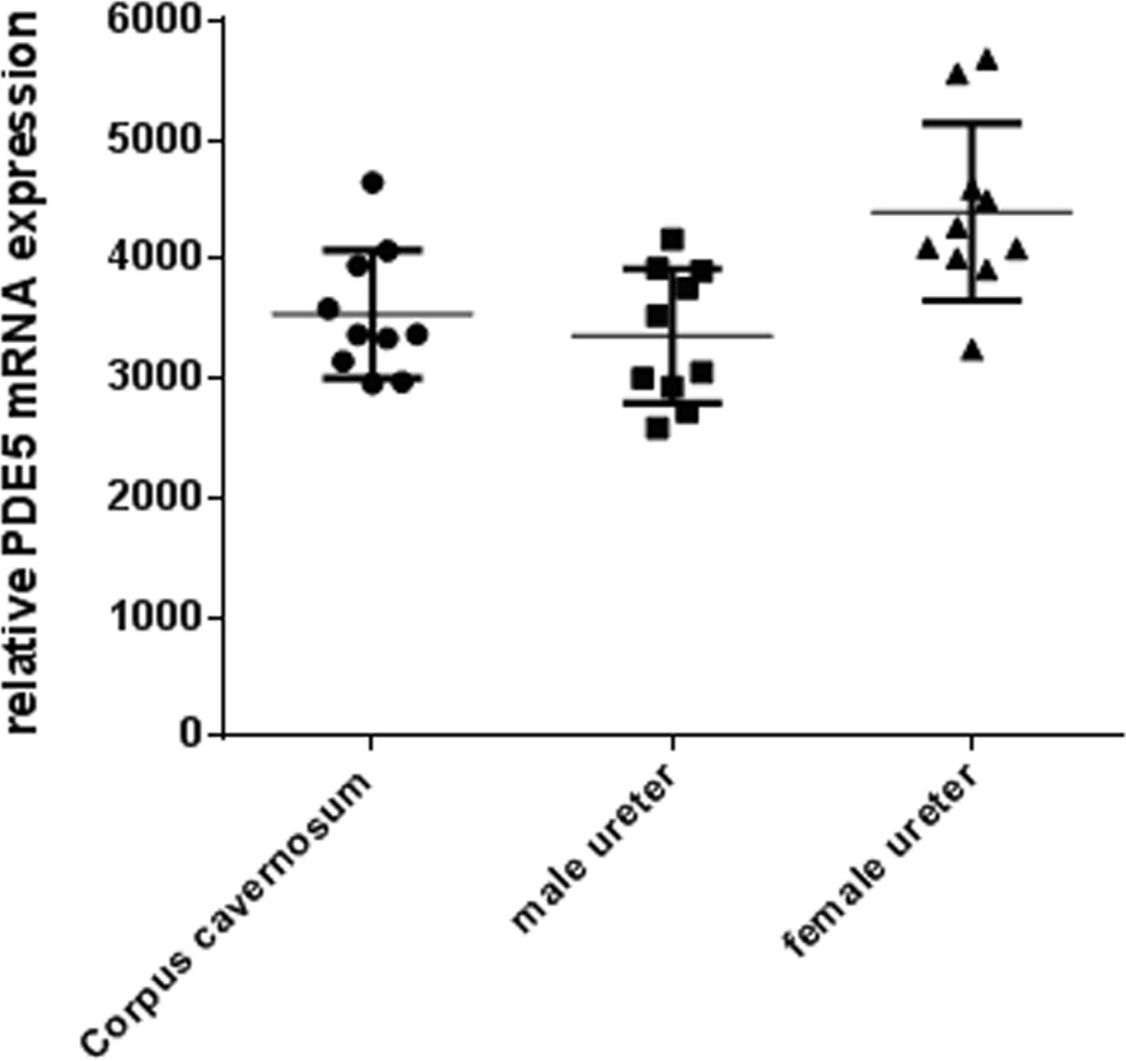
PDE-5 mRNA expression in corpus cavernosum and ureter. Relative PDE-5 mRNA expression in the corpus cavernosum and male and female ureter in rats. Data are mean ± SD, n = 10.

PDE5 mRNA expression was quantified with RT-PCR in corpus cavernosum and in ureters of both sexes. PDE5 mRNA was abundantly expressed in the ureter of both sexes on a similar range as in corpus cavernosum. The expression tended to be slightly higher in the female ureter (Fig 1), without, however, reaching statistical significance. This substantial and not sex-specific expression in rat ureter suggests a functional impact of PDE5 in males and females.

### Ureteral contractility studies

**Fig 2:**
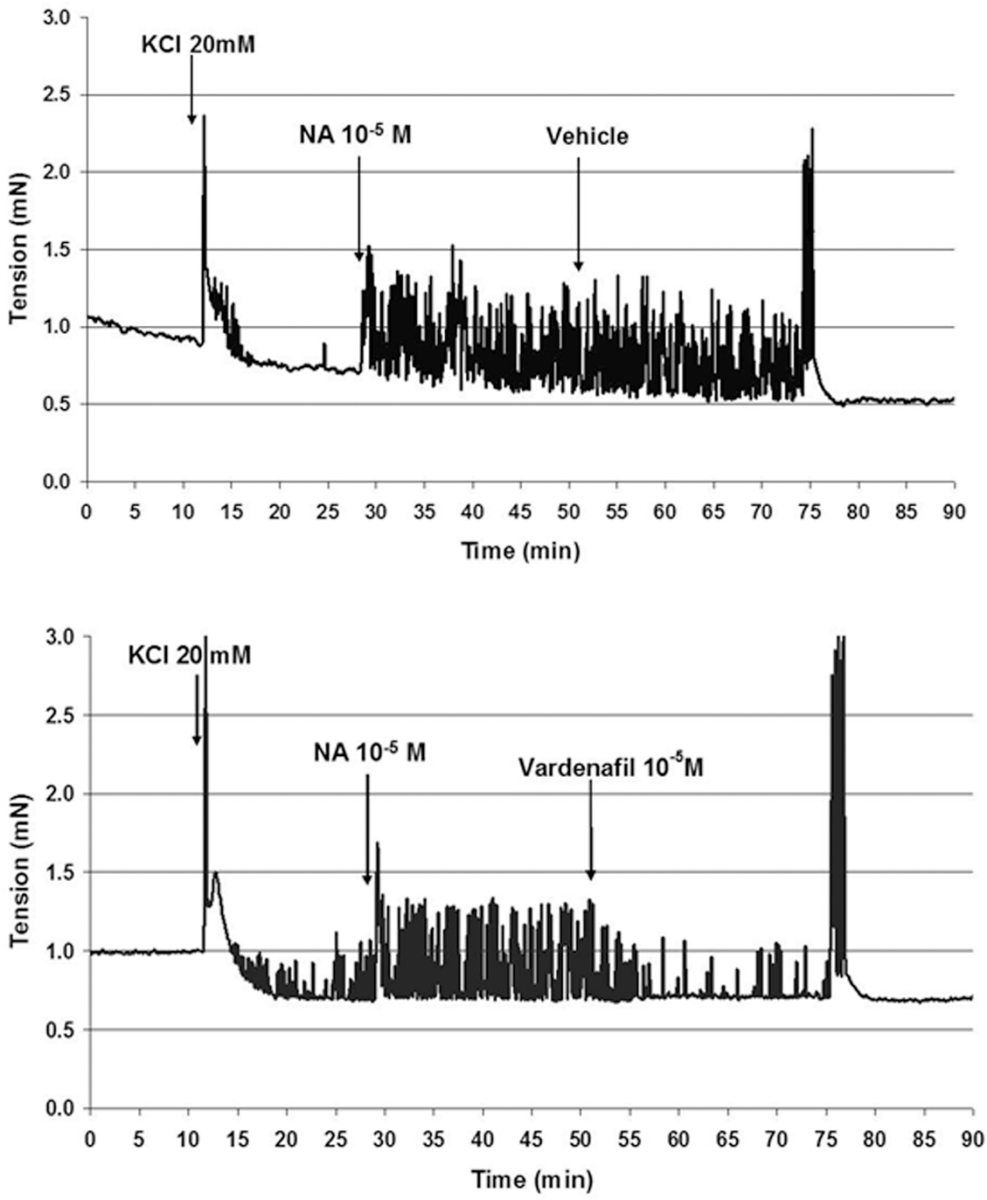
Contractility of isolated ureters. Noradrenaline (10μM)—induced oscillations of isolated rat ureter (upper panel) which can be blocked by vardenafil (10μM) (lower panel).

Representative original recordings of force from isolated rat ureters are shown in Fig 2. High potassium (20mM) induced spontaneous phasic contractions. When noradrenaline (10μM) was added, the preparations became spontaneously active. Addition of the vehicle was not able to reduce noradrenaline-induced spontaneous activity (Fig 2, upper panel). The application of vardenafil (10μM) reduced the frequency of the oscillations (Fig 2, lower panel).

**Fig 3:**
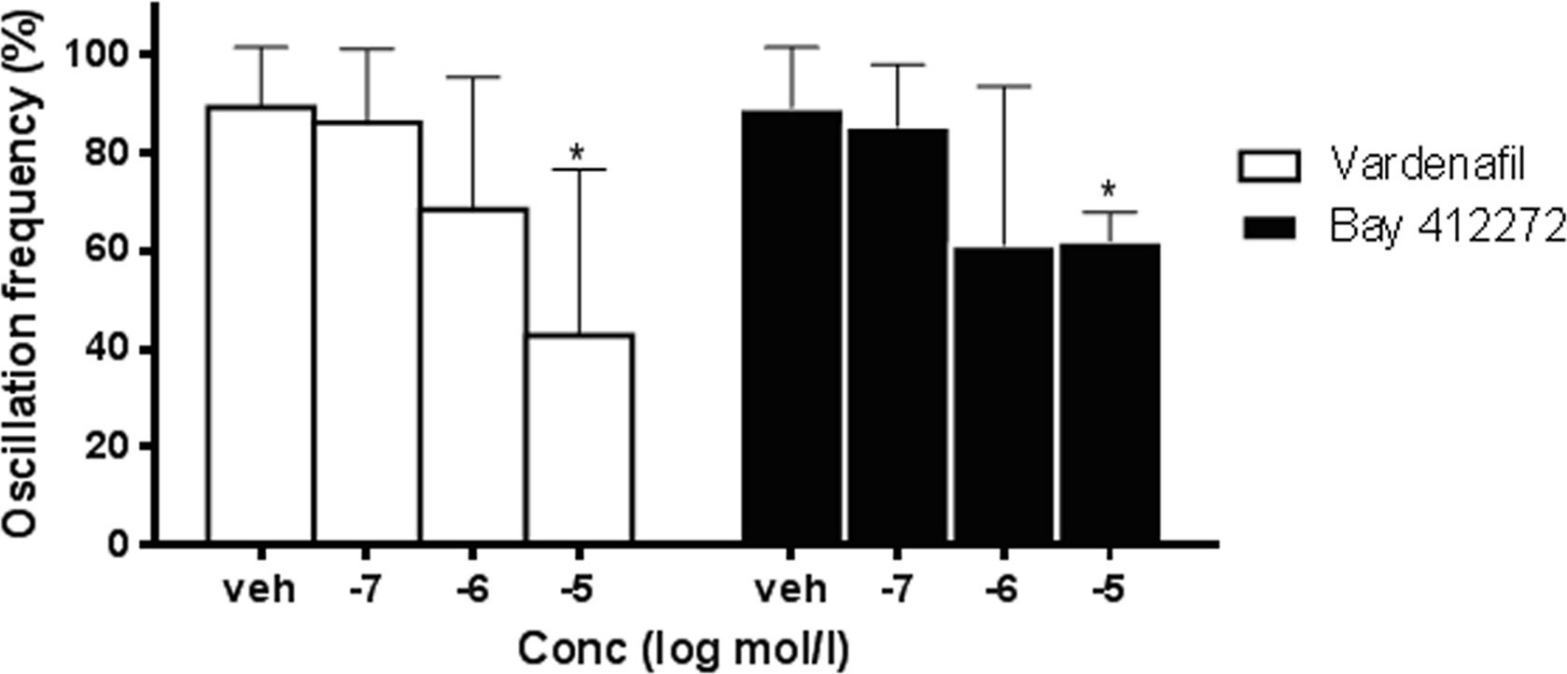
Contractility of isolated ureters. Effects of the PDE5 inhibitor vardenafil and the sGC stimulator BAY 41–2272 on noradrenaline-induced oscillations of isolated rat ureter compared to vehicle. Data are presented as Mean ± SD, n = 4–8; *p<0.05 vs. vehicle (S1 File).

The application of the sGC stimulator BAY 41–2272 (10μM) also reduced the frequency of noradrenaline-induced oscillations of rat ureters. The results with the PDE5 inhibitor vardenafil and the sGC stimulator BAY 41–2272 on ureteral contractility are summarized in Fig 3 (S1 File). Both vardenafil and BAY 41–2272 caused a concentration-dependent and significant reduction of spontaneous oscillations when compared to the vehicle group. The efficacy of vardenafil and the sGC stimulator BAY 41–2272 were in a similar range and not significantly different at 1 and 10μM.

### Behavioural study

#### Ureteral crises

**Fig 4:**
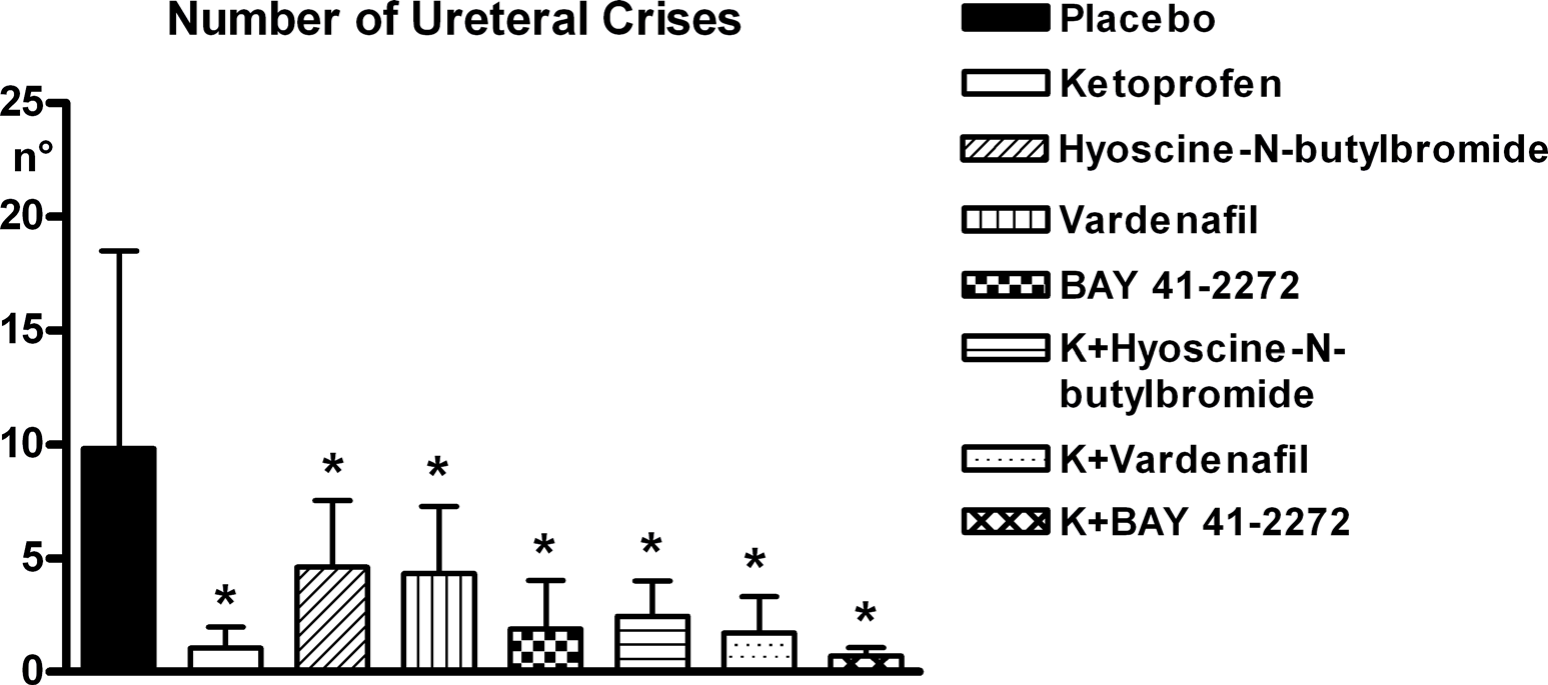
Ureteral pain behaviour. Number of crises over 4 days after stone implantation in the various experimental groups (18 rats per group; Means ± SD). 1-way ANOVA: significant trend; asterisks over SD bars refer to comparison between each experimental group and the placebo group. * = p<0.01 (S2 File).

**Fig 5:**
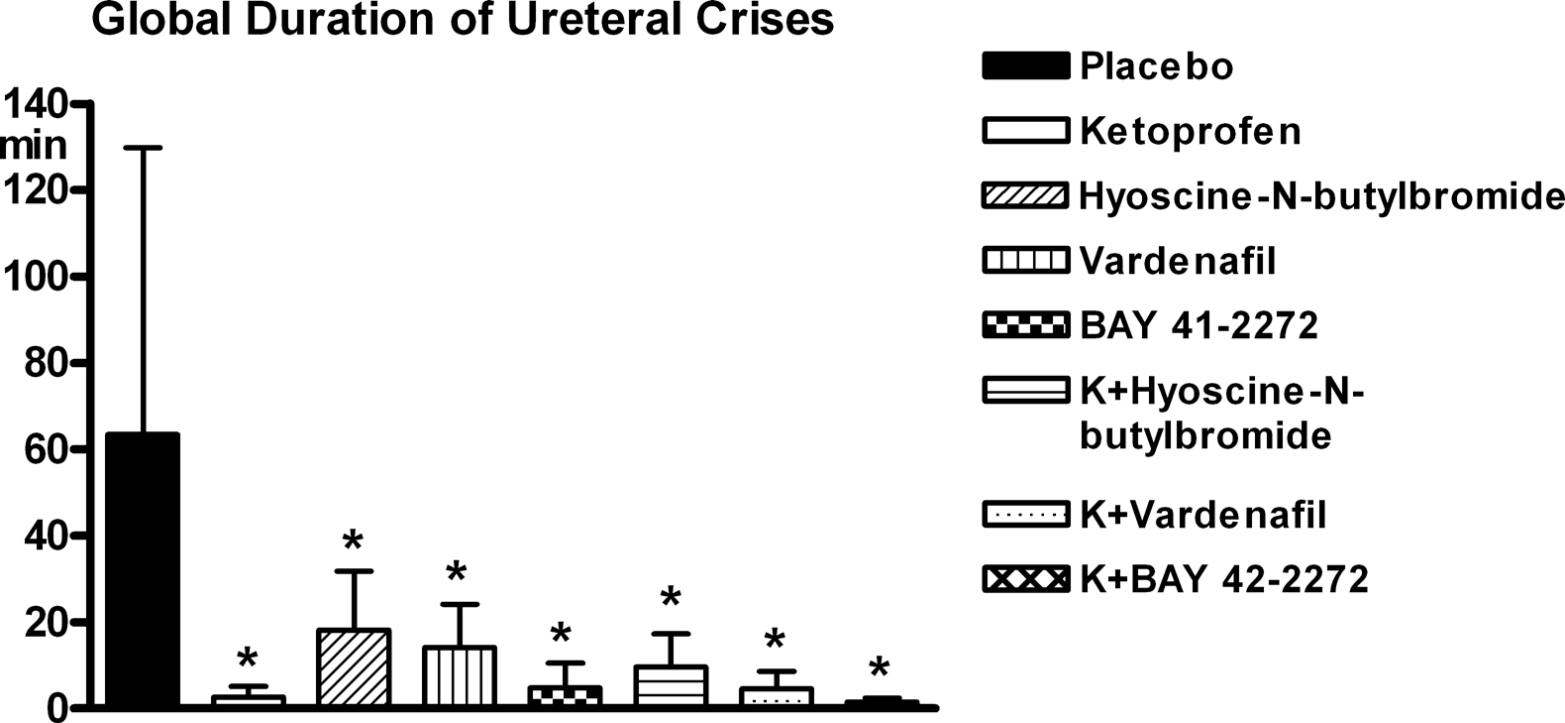
Ureteral pain behaviour. Global duration of crises. Legend as for Fig 4 (S2 File).

**Fig 6:**
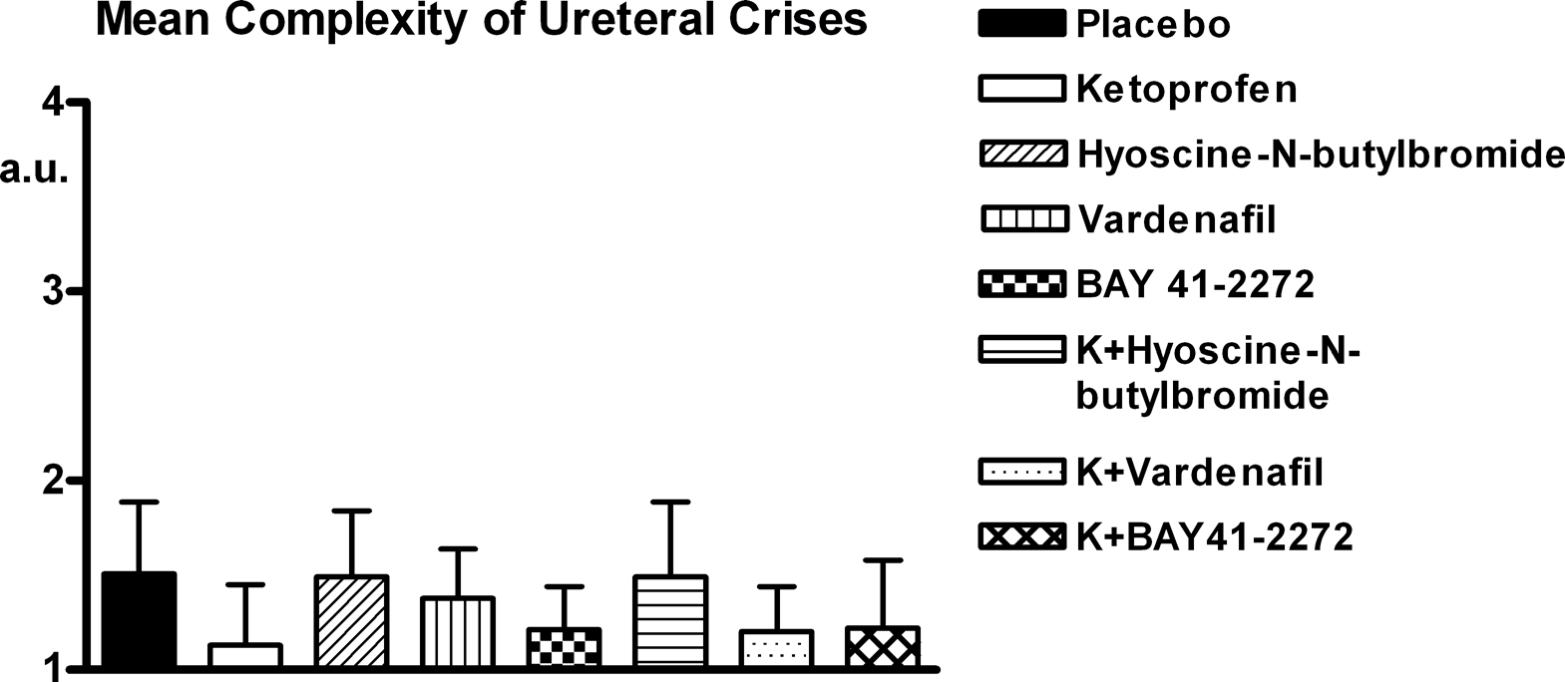
Ureteral pain behaviour. Mean complexity of crises. Legend as for Fig 4 (S2 File).

The parameters of the crises are reported in Figs 4–6 (S2 File). All experimental compounds significantly reduced the number and global duration of the crises. Among the three spasmolytics administered alone, the compound producing the highest level of reduction was BAY 41–2272, while the combination of ketoprofen with BAY 41–2272 determined the highest effect in absolute, although none of these differences among compounds was significant. The complexity of the crises underwent less pronounced changes with treatment, none of which was significant.

#### Muscle sensitivity

**Fig 7:**
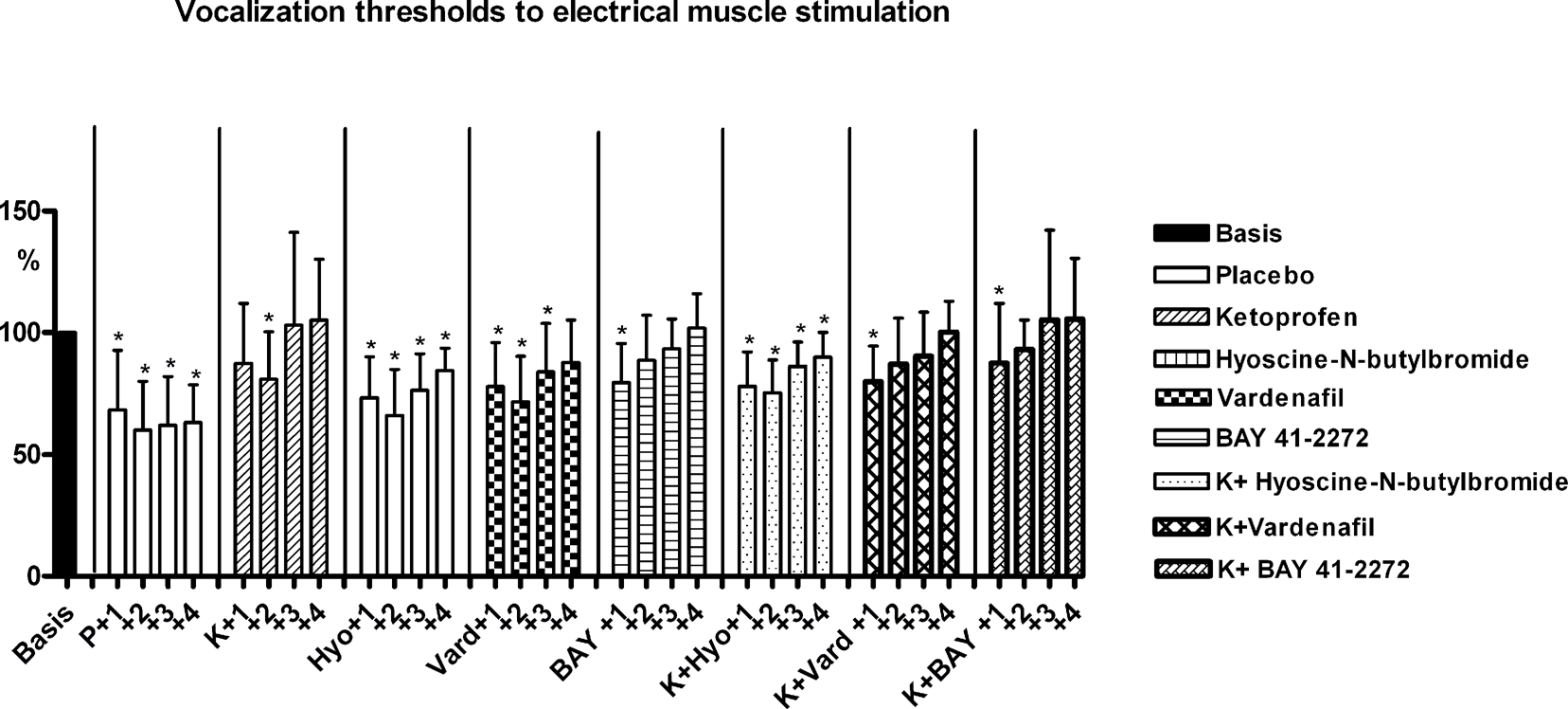
Muscle hyperalgesia. Vocalization thresholds to electrical stimulation in the various experimental groups. Thresholds over 4 days post-stone implantation (+1,+2,+3 and +4) are expressed as a percentage of pre-operative values (Basis = mean of thresholds on days: -2,-1 and 0 = 100%)(18 rats per group, Means ± SD). ANOVA for repeated measures: significant trend; in each group, asterisks over SD bars refer to the comparison between values of each day and basal values. * p<0.01 (S3 File).

The results of vocalization thresholds to electrical stimulation of the left oblique muscle are reported in Fig 7 (S3 File). All experimental compounds significantly reduced the post-stone vocalization threshold decrease (muscle hyperalgesia). The spasmolytic producing the highest reduction of muscle hyperalgesia, when administered alone, was BAY 41–2272; it was less effective than ketoprofen alone on day 1, but more effective than ketoprofen on day 2. The most pronounced hyperalgesia reduction in the combination regimens was observed with ketoprofen plus BAY 41–2272.

### Autopsy findings

Table 1 summarizes the percentage of stone elimination at autopsy in each experimental group of rats. Except vardenafil alone, all experimental compounds promoted a higher stone elimination with respect to placebo, with a two-fold increase by BAY 41–2272 alone (similarly to ketoprofen, and higher than hyoscine-N-butylbromide) and a three-fold increase by ketoprofen + BAY 41–2272. The differences among groups did not, however, reach statistical significance with the relatively small sample sizes.

**Table 1:** Stone elimination.

Experimental group	Number and % of rats
Placebo	2 rats (11%)
Ketoprofen	5 rats (28%)
Hyoscine-N-butylbromide	3 rats (22%)
Vardenafil	2 rats (11%)
BAY 41–2272	4 rats (22%)
K + Hyoscine-N-butylbromide	5 rats (33%)
K + Vardenafil	3 rats (17%)
K + BAY 41–2272	7 rats (39%)

Percentage of stone elimination in the experimental groups (18 rats each)

## Discussion and Conclusions

The results of the present study show that PDE5 mRNA is abundantly expressed in the ureter of male and female rats and expression levels in ureteral tissues are as high as in corpus cavernosum in which PDE5 plays a significant functional role. These data reconfirm previous work showing PDE5 expression in the ureter of other species [19,20]. In addition, it has been shown in previous studies in the rat, that nitric oxide synthase (NOS)-containing nerves are present in the mucosal stroma, that these nerves are encircling small arteries of the urethra and are also found at the border of the smooth muscle layers and in close proximity to the urothelium [37]. Moreover, also a significant guanylate cyclase activity was detected in rat ureteral tissue [38]. Expression of the soluble guanylate cyclase was found in the smooth muscle and urothelium layer and expression of PDE5 in the smooth muscle only [39]. All together, these descriptive data demonstrated that the NO/cGMP/PDE5 system is present in ureteral tissues of different species and pointed towards a functional role of this common pathway. Therefore, we performed functional contractility studies of isolated ureteral tissues. In the organ bath-assay *in vitro* we demonstrated that the PDE5 inhibitor vardenafil, but also the sGC stimulator BAY 41–2272, significantly and dose-dependently reduced noradrenaline-induced spontaneous oscillations of isolated rat ureter. These data are in line with the recently published data of Kedia et al (2013), Liatsikos et al (2013), and Miyaoka et al (2014)[39,40,41], showing a significant and concentration-dependent relaxation in human ureteral tissue with the NO-donnor sodiumnitroprusside (SNP), the PDE5 inhibitors vardenafil and sildenafil [40,41] and the sGC stimulator BAY 41–2272 [39]. In human ureteral tissue, the relaxing effects of vardenafil were also accompanied by cGMP increase, which suggests a cGMP-driven mode of action [41]. In our studies, the efficacy of both vardenafil and BAY 41–2272 on ureteral contractility were in a similar range and significant over placebo at 10μM, which is also in line with the previous data [39,41]. Since the organ bath experiments are done in healthy tissue, this may not allow discrimination between the different mechanisms. However, these data in rat and human tissue strongly suggest that the NO/cGMP pathway is expressed in ureteral tissues throughout species and plays a functional role in the regulation of the ureteral tone. Despite these *in vitro* effects, however, to the best of our knowledge no *in vivo* studies have so far been published to confirm the *in vivo* relevance of these observations. Thus, we wanted to investigate if the ureteral expression of the NO/cGMP pathway and the relaxing effects of PDE5 inhibitors and sGC stimulators translate into a meaningful benefit *in vivo*. Therefore, we tested if the treatment with vardenafil and BAY 41–2272 has an impact on the ureteral pain behaviour/referred muscle hyperalgesia and on stone expulsion in an animal model of ureteral calculosis. Both vardenafil and BAY 41–2272 administered alone significantly reduced behavioural indicators of colic pain and referred muscle hyperalgesia with respect to placebo. This result confirms the impact of ureteral hypercontractility in the genesis of urinary colic pain from calculosis. Although none of the inter-treatment comparisons were statistically significant, numerical trends were found in the extent of reduction of pain behaviour with the various compounds. BAY 41–2272 had more pronounced effects than vardenafil and the classic spasmolytic hyposcine-N-butylbromide. Furthermore, the combination of BAY 41–2272 with ketoprofen produced the highest reduction of the behaviour, in parallel with the highest percentage of stone expulsion, which suggests that the anti-inflammatory and spasmolytic effects combine to promote stone progression along the ureter.

Our *in vivo* data extend previous *in vitro* findings from organ bath experiments in which cGMP increase via PDE5 inhibitors and sGC stimulators was able to relax ureteral tone. Moreover, our data suggest for the first time *in vivo* that PDE5 inhibitors and sGC stimulators alone but especially in combination with NSAIDs facilitate stone expulsion and reduce colic pain. However, despite the observed trends, future studies with potentially increased sample sizes would be indicated in order for final conclusions to be drawn on the promotion of stone expulsion.

It might also be interesting to investigate in future studies how cGMP effects in such kinds of *in vivo* models compare with effects of alpha1 antagonists, which are already in use clinically for stone expulsion [12]. This is also interesting since it has been found that alpha blockers fasten stone expulsion but have little effect on overall expulsion rate. Therefore it would be worthwhile investigating if cGMP mediated treatment approaches are here different.

In conclusion, the present results show that the NO/cGMP/PDE5 pathway is involved in the regulation of ureteral contractility and stone expulsion in urinary calculosis. PDE5 inhibitors and especially sGC stimulators, also in combination with a classic NSAID, could become a potent new treatment option, to reduce colic pain related to urinary calculosis.

## Supporting Information

S1 File Raw data of Fig 3. (PZF)

S2 File Raw data of Figs 4, 5 and 6. (DOC)

S3 File Raw data of Fig 7. (DOC)

## Abbreviations

cGMP: cyclic guanosine monophosphate
ESWL: extracorporeal shock wave lithotripsy
NO: Nitric Oxide
PDE5: phosphodiesterase type 5
sGC: soluble guanylate cyclase

## References

[1] Giamberardino MA, Affaitati G, Costantini R. Chapter 24. Referred pain from internal organs. Handb Clin Neurol. 2006;81:343–361. 18808846 10.1016/S0072-9752(06)80028-X

[2] Giamberardino MA, Costantini R, Affaitati G, Fabrizio A, Lapenna D, Tafuri E, et al Viscero-visceral hyperalgesia: characterization in different clinical models. Pain. 2010;151:307–322. 10.1016/j.pain.2010.06.023 20638177

[3] Trinchieri A. Epidemiology of urolithiasis: an update. Clin Cases Miner Bone Metab. 2008; 5(2):101–106. 22460989 PMC2781200

[4] Tseng TY, Stoller ML. Medical and medical/urologic approaches in acute and chronic urologic stone disease. Med Clin North Am. 2011; 95(1):169–177. 10.1016/j.mcna.2010.08.034 21095420

[5] Giamberardino MA, de Bigontina P, Martegiani C, Vecchiet L. Effects of extracorporeal shock-wave lithotripsy on referred hyperalgesia from renal/ureteral calculosis. Pain. 1994; 56(1):77–83. 8159443 10.1016/0304-3959(94)90152-X

[6] Vecchiet L, Giamberardino MA, de Bigontina P. Referred pain from viscera: when the symptom persists despite the extinction of the visceral focus. Adv Pain Res Ther. 1992; 20:101–110.

[7] Vecchiet L, Giamberardino MA, Dragani L, Albe-Fessard D. Pain from renal/ureteral calculosis: evaluation of sensory thresholds in the lumbar area. Pain. 1989; 36: 289–295. 2710558 10.1016/0304-3959(89)90087-0

[8] Bultitude M, Rees J. Management of renal colic. BMJ. 2012; 345:e5499 10.1136/bmj.e5499 22932919

[9] Macneil F, Bariol S. Urinary stone disease—assessment and management. Aust Fam Physician. 2011; 40(10):772–725. 22003478

[10] Canda AE, Turna B, Cinar GM, Nazli O. Physiology and pharmacology of the human ureter: basis for current and future treatments. Urol Int. 2007; 78:289–298. 17495484 10.1159/000100830

[11] Singh A, Alter HJ, Littlepage A. A systematic review of medical therapy to facilitate passage of ureteral calculi. Ann Emerg Med. 2007; 50(5):552–563. 17681643 10.1016/j.annemergmed.2007.05.015

[12] Zhu Y, Duijvesz D, Rovers MM, Lock TM. Alpha-Blockers to assist stone clearance after extracorporeal shock wave lithotripsy: a meta-analysis. BJU Int. 2010; 106(2):256–261. 10.1111/j.1464-410X.2009.09014.x 19889063

[13] Albersen M, Linsen L, Tinel H, Sandner P, Van Renterghem K. Synergistic effects of BAY 60–4552 and vardenafil on relaxation of corpus cavernosum tissue of patients with erectile dysfunction and clinical phosphodiesterase type 5 inhibitor failure. J Sex Med. 2013; 10:1268–1277. 10.1111/jsm.12095 23421435

[14] Lin CS, Albersen M, Xin Z, Namiki M, Muller D, Lue TF. Phosphodiesterase-5 expression and function in the lower urinary tract: a critical review. Urology. 2013; 81(3):480–487. 10.1016/j.urology.2012.11.028 23333001 PMC3839665

[15] Martínez-Salamanca JI, Carballido J, Eardley I, Giuliano F, Gratzke C, Rosen R, et al Phosphodiesterase type 5 inhibitors in the management of non-neurogenic male lower urinary tract symptoms: critical analysis of current evidence. Eur Urol. 2011; 60(3):527–535. 10.1016/j.eururo.2011.05.054 21684677

[16] Miller MS. Role of phosphodiesterase type 5 inhibitors for lower urinary tract symptoms. Ann Pharmacother. 2013; 47(2):278–283. 10.1345/aph.1R528 23386068

[17] Filippi S, Morelli A, Sandner P, Fibbi B, Mancina R, Marini M, et al Characterization and functional role of androgen-dependent PDE5 activity in the bladder. Endocrinology. 2007; 148:1019–1029. 17138653 10.1210/en.2006-1079

[18] Tinel H, Stelte-Ludwig B, Hütter J, Sandner P. Pre-clinical evidence for the use of phosphodiesterase-5 inhibitors for treating benign prostatic hyperplasia and lower urinary tract symptoms. BJU Int. 2006; 98(6):1259–1263. 16956354 10.1111/j.1464-410X.2006.06501.x

[19] Gratzke C, Uckert S, Kedia G, Reich O, Schlenker B, Seitz M, et al In vitro effects of PDE5 inhibitors sildenafil, vardenafil and tadalafil on isolated human ureteral smooth muscle: a basic research approach. Urol Res. 2007; 35(1):49–54. 17102958 10.1007/s00240-006-0073-1

[20] Taher A, Schulz-Knappe P, Meyer M, Truss M, Forssmann WG, Stief CG, et al Characterization of cyclic nucleotide phosphodiesterase isoenzymes in the human ureter and their functional role in vitro. World J Urol. 1994; 12(5):286–291. 7866426 10.1007/BF00191209

[21] Stasch JP, Pacher P, Evgenov OV. Soluble guanylate cyclase as an emerging therapeutic target in cardiopulmonary disease. Circulation. 2011; 123(20):2263–2273. 10.1161/CIRCULATIONAHA.110.981738 21606405 PMC3103045

[22] Giamberardino MA, Valente R, de Bigontina P, Vecchiet L. Artificial ureteral calculosis in rats: behavioural characterization of visceral pain episodes and their relationship with referred lumbar muscle hyperalgesia. Pain. 1995a; 61(3):459–469. 7478690 10.1016/0304-3959(94)00208-V

[23] Giamberardino MA, Tana C, Costantini R. Pain thresholds in women with chronic pelvic pain. Curr Opin Obstet Gynecol. 2014;26(4):253–259. 10.1097/GCO.0000000000000083 24921647

[24] Affaitati G, Giamberardino MA, Lerza R, Lapenna D, De Laurentis S, Vecchiet L. Effects of tramadol on behavioural indicators of colic pain in a rat model of ureteral calculosis. Fundam Clin Pharmacol. 2002; 16:23–30. 11903509 10.1046/j.1472-8206.2002.00068.x

[25] Giamberardino MA, Affaitati G, Lerza R, Vecchiet L. Pre-emptive analgesia in rats with artificial ureteric calculosis. Effects on visceral pain behavior in the post-operative period. Brain Res. 2000;878(1–2):148–54. 10996145 10.1016/s0006-8993(00)02728-1

[26] Giamberardino MA, Valente R, De Bigontina P, Iezzi S, Vecchiet L. Effects of spasmolytic and/or non-steroidal antiinflammatory drugs on muscle hyperalgesia of ureteral origin in rats. Eur J Pharmacol. 1995b; 278(2):97–101. 7672006 10.1016/0014-2999(95)00104-s

[27] Iuvone T, Affaitati G, De Filippis D, Lopopolo M, Grassia G, Lapenna D, et al Ultramicronized palmitoylethanolamide reduces viscero-visceral hyperalgesia in a rat model of endometriosis plus ureteral calculosis: role of mast cells. Pain. 2015 5 6. [Epub ahead of print] 10.1097/j.pain.0000000000000220 25974242

[28] Laird JM, Roza C, Olivar T. Antinociceptive activity of metamizol in rats with experimental ureteric calculosis: central and peripheral components. Inflamm Res. 1998; 47(10):389–395. 9831323 10.1007/s000110050349

[29] Lopopolo M, Affaitati G, Fabrizio A, Massimini F, Lapenna D, Giamberardino MA, et al Effects of tramadol on viscero-visceral hyperalgesia in a rat model of endometriosis plus ureteral calculosis. Fundam Clin Pharmacol. 2014;28(3):331–341. 10.1111/fcp.12038 23786290

[30] Laird JM, Roza C, Cervero F. Effects of artificial calculosis on rat ureter motility: peripheral contribution to the pain of ureteric colic. Am J Physiol. 1997; 272(5 Pt 2):R1409–1416. 9176331 10.1152/ajpregu.1997.272.5.R1409

[31] Tinel H, Stelte-Ludwig B, Hütter J, Sandner P. Pre-clinical evidence for the use of phosphodiesterase-5 inhibitors for treating benign prostatic hyperplasia and lower urinary tract symptoms. BJU Int. 2006;98(6):1259–63. 16956354 10.1111/j.1464-410X.2006.06501.x

[32] Mulvany MJ, Halpern W. Contractile properties of small arterial resistance vessels in spontaneously hypertensive and normotensive rats. Circ Res. 1977;41(1):19–26. 862138 10.1161/01.res.41.1.19

[33] Affaitati G, Ceccarelli I, Fiorenzani P, Rossi C, Pace MC, Passavanti MB, et al Sex differences in the analgesic effects of ICI 182,780 and Flutamide on ureteral calculosis in rats. Horm Behav. 2011; 59:9–13. 10.1016/j.yhbeh.2010.09.008 20920504

[34] Aloisi AM, Affaitati G, Ceccarelli I, Fiorenzani P, Lerza R, Rossi C, et al Estradiol and testosterone differently affect visceral pain-related behavioural responses in male and female rats. Eur J Pain. 2010; 14:602–607. 10.1016/j.ejpain.2009.10.016 19948419

[35] Kilkenny C, Browne W, Cuthill IC, Emerson M, Altman DG. Animal research: reporting in vivo experiments: The ARRIVE guidelines. BJP. 2010; 160: 1577–1579. 10.1111/j.1476-5381.2010.00872.x PMC2936830 20649561

[36] Albe-Fessard D, Giamberardino MA, Rampin O. Comparisons of different animal models of chronic pain. Adv Pain Res Ther. 1990; 13:11–27.

[37] Alm P, Larsson B, Ekblad E, Sundler F, Andersson KE. Immunohistochemical localization of peripheral nitric oxide synthase-containing nerves using antibodies raised against synthesized C- and N-terminal fragments of a cloned enzyme from rat brain. Acta Physiol Scand. 1993; 148:421–429. 7692702 10.1111/j.1748-1716.1993.tb09578.x

[38] Ehrén I, Iversen H, Jansson O, Adolfsson J, Wiklund NP. Localization of nitric oxide synthase activity in the human lower urinary tract and its correlation with neuroeffector responses. Urology. 1994; 44:683–687. 7526524 10.1016/s0090-4295(94)80206-8

[39] Miyaoka R, Mendes C, Schenka A, Gonzalez PG, de Nucci G, Antunes E, et al BAY 41–2272, a Soluble Guanylate Cyclase Stimulator, Relaxes Isolated Human Ureter in a Standardized In Vitro Model. Urology. 2014; 83(1):256.e1–7. 10.1016/j.urology.2013.09.005 24231204

[40] Kedia GT, Oelke M, Sohn M, Kuczyk MA, Ückert S. Pharmacologic characterization of human male urethral smooth muscle: an in vitro approach. Urology. 2013; 82(6):1451.e13–9 10.1016/j.urology.2013.08.023 24295263

[41] Liatsikos E, Kyriazis I, Neuhaus J, Kallidonis P, Georgiopoulos I, Franz T, et al Direct effects of vardenafil on the ureter: in vitro investigation and potential clinical applications of intralumenal administration. J Endourol. 2013; 27(11):1400–1404. 10.1089/end.2012.0612 23763505

