# Supplementary material for: Effects of PDE5 Inhibitors and sGC Stimulators in a Rat Model of Artificial Ureteral Calculosis
Source: PLoS One. 2015 Oct 28;10(10):e0141477. doi: 10.1371/journal.pone.0141477 (PMC4624930; doi:10.1371/journal.pone.0141477)
Supplement: S2 File — (DOC) [file pone.0141477.s002.doc]

**Ureteral Pain Behaviour**

| **Placebo Number** | **Placebo**  **Duration** | **Placebo**  **Complexity** |  | **Ketoprofen**  **Number** | **Ketoprofen**  **Duration** | **Ketoprofen**  **Complexity** |  | **Hyoscine**  **Number** | **Hyoscine**  **Duration** | **Hyoscine**  **Complexity** |
| --- | --- | --- | --- | --- | --- | --- | --- | --- | --- | --- |
| 2 | 5 | 2.30 |  | 1 | 3 | 1.0 |  | 1 | 2 | 1.00 |
| 10 | 85 | 2.00 |  | 2 | 7 | 2.0 |  | 6 | 21 | 1.50 |
| 7 | 39 | 1.67 |  | 0 | 0 |  |  | 3 | 7 | 1.00 |
| 3 | 14 | 1.20 |  | 2 | 4 | 1.0 |  | 4 | 17 | 1.75 |
| 5 | 49 | 1.00 |  | 0 | 0 |  |  | 2 | 8 | 2.00 |
| 3 | 10 | 1.22 |  | 0 | 0 |  |  | 4 | 9 | 1.25 |
| 9 | 42 | 1.00 |  | 1 | 2 | 1.0 |  | 3 | 15 | 1.15 |
| 12 | 41 | 1.54 |  | 2 | 4 | 1.0 |  | 7 | 55 | 1.83 |
| 7 | 43 | 1.75 |  | 2 | 6 | 1.0 |  | 6 | 14 | 1.25 |
| 9 | 37 | 1.50 |  | 2 | 4 | 1.0 |  | 12 | 35 | 2.14 |
| 4 | 46 | 1.14 |  | 0 | 0 |  |  | 2 | 8 | 1.50 |
| 6 | 22 | 1.75 |  | 0 | 0 |  |  | 4 | 22 | 1.83 |
| 7 | 55 | 1.33 |  | 1 | 3 | 1.0 |  | 5 | 24 | 1.33 |
| 16 | 128 | 1.91 |  | 2 | 4 | 1.0 |  | 3 | 10 | 1.25 |
| 38 | 267 | 1.10 |  | 2 | 5 | 1.5 |  | 2 | 7 | 1.25 |
| 10 | 60 | 1.78 |  | 2 | 6 | 1.0 |  | 11 | 41 | 1.91 |
| 23 | 179 | 1.17 |  | 0 | 0 |  |  | 4 | 18 | 1.25 |
| 6 | 20 | 1.89 |  | 0 | 0 |  |  | 4 | 15 | 1.75 |

| **Vardenafil**  **Number** | **Vardenafil**  **Duration** | **Vardenafil**  **Complexity** |  | **BAY**  **Number** | **BAY**  **Duration** | **BAY**  **Complexity** |  | **Keto+Hyo**  **Number** | **Keto+Hyo Duration** | **Keto+Hyo Complexity** |
| --- | --- | --- | --- | --- | --- | --- | --- | --- | --- | --- |
| 4 | 15 | 1.25 |  | 1 | 2 | 1.00 |  | 5 | 25 | 1.75 |
| 9 | 40 | 1.55 |  | 2 | 4 | 1.00 |  | 2 | 7 | 1.50 |
| 8 | 29 | 1.62 |  | 4 | 14 | 1.00 |  | 0 | 0 |  |
| 2 | 6 | 1.00 |  | 0 | 0 |  |  | 2 | 5 | 1.00 |
| 3 | 10 | 1.33 |  | 0 | 0 |  |  | 6 | 27 | 2.00 |
| 7 | 24 | 1.28 |  | 0 | 0 |  |  | 3 | 6 | 1.00 |
| 1 | 2 | 1.00 |  | 4 | 11 | 1.50 |  | 1 | 3 | 1.15 |
| 5 | 16 | 1.40 |  | 7 | 18 | 1.28 |  | 1 | 4 | 1.50 |
| 0 | 0 |  |  | 2 | 7 | 1.50 |  | 3 | 19 | 1.75 |
| 4 | 8 | 1.25 |  | 0 | 0 |  |  | 3 | 13 | 1.50 |
| 5 | 17 | 1.40 |  | 5 | 10 | 1.00 |  | 1 | 2 | 1.00 |
| 7 | 19 | 1.85 |  | 0 | 0 |  |  | 2 | 8 | 1.75 |
| 5 | 13 | 1.40 |  | 0 | 0 |  |  | 5 | 15 | 2.00 |
| 3 | 11 | 1.33 |  | 3 | 10 | 1.33 |  | 3 | 14 | 1.75 |
| 7 | 18 | 2.00 |  | 2 | 4 | 1.00 |  | 2 | 9 | 1.00 |
| 4 | 14 | 1.25 |  | 4 | 8 | 1.50 |  | 1 | 5 | 1.00 |
| 0 | 0 |  |  | 0 | 0 |  |  | 2 | 4 | 1.00 |
| 4 | 13 | 1.25 |  | 0 | 0 |  |  | 2 | 7 | 2.00 |

| **Keto+Vard**  **Number** | **Keto+Vard**  **Duration** | **Keto+Vard**  **Complexity** |  | **Keto+BAY**  **Number** | **Keto+BAY**  **Duration** | **Keto+BAY**  **Complexity** |
| --- | --- | --- | --- | --- | --- | --- |
| 2 | 5 | 1.5 |  | 0 | 0 |  |
| 1 | 2 | 1.0 |  | 0 | 0 |  |
| 0 | 0 |  |  | 0 | 0 |  |
| 1 | 2 | 1.0 |  | 0 | 0 |  |
| 2 | 7 | 1.5 |  | 2 | 5 | 1.0 |
| 2 | 6 | 1.0 |  | 1 | 2 | 1.0 |
| 0 | 0 |  |  | 0 | 0 |  |
| 6 | 15 | 1.5 |  | 2 | 4 | 1.0 |
| 2 | 6 | 1.5 |  | 0 | 0 |  |
| 1 | 3 | 1.0 |  | 0 | 0 |  |
| 2 | 6 | 1.0 |  | 0 | 0 |  |
| 0 | 0 |  |  | 1 | 2 | 1.0 |
| 2 | 7 | 1.5 |  | 2 | 5 | 1.5 |
| 2 | 5 | 1.0 |  | 1 | 2 | 1.0 |
| 0 | 0 |  |  | 0 | 0 |  |
| 5 | 12 | 1.4 |  | 2 | 4 | 1.5 |
| 2 | 4 | 1.0 |  | 1 | 2 | 1.0 |
| 1 | 3 | 1.0 |  | 1 | 3 | 2.0 |
