# Supplementary material for: Effects of PDE5 Inhibitors and sGC Stimulators in a Rat Model of Artificial Ureteral Calculosis
Source: PLoS One. 2015 Oct 28;10(10):e0141477. doi: 10.1371/journal.pone.0141477 (PMC4624930; doi:10.1371/journal.pone.0141477)
Supplement: S3 File — (DOC) [file pone.0141477.s003.doc]

**Vocalization thresholds to electrical muscle stimulation**

| **Basis** | **P+1** | **P+2** | **P+3** | **P+4** |
| --- | --- | --- | --- | --- |
| 100 | 97.95 | 105.10 | 84.28 | 91.83 |
| 100 | 37.50 | 45.31 | 47.18 | 50.31 |
| 100 | 53.77 | 58.12 | 90.84 | 67.50 |
| 100 | 99.03 | 71.42 | 72.39 | 65.37 |
| 100 | 80.48 | 52.68 | 55.60 | 60.97 |
| 100 | 81.75 | 82.50 | 92.50 | 92.00 |
| 100 | 60.00 | 62.85 | 65.71 | 68.57 |
| 100 | 97.61 | 50.00 | 26.46 | 52.91 |
| 100 | 65.32 | 57.78 | 61.30 | 67.33 |
| 100 | 71.79 | 68.97 | 69.48 | 67.94 |
| 100 | 84.55 | 51.12 | 62.35 | 65.16 |
| 100 | 73.33 | 82.66 | 76.66 | 76.66 |
| 100 | 101.29 | 38.63 | 40.58 | 51.62 |
| 100 | 35.52 | 52.36 | 50.78 | 49.21 |
| 100 | 30.14 | 31.63 | 32.48 | 44.58 |
| 100 | 46.50 | 57.00 | 59.25 | 50.75 |
| 100 | 34.24 | 27.29 | 39.70 | 34.73 |
| 100 | 81.08 | 86.48 | 88.91 | 80.50 |

| **K+1** | **K+2** | **K+3** | **K+4** |
| --- | --- | --- | --- |
| 72.07 | 67.52 | 65.24 | 95.44 |
| 65.57 | 44.47 | 98.99 | 95.72 |
| 92.30 | 79.48 | 115.38 | 97.43 |
| 62.25 | 62.00 | 95.00 | 95.00 |
| 82.50 | 92.22 | 127.77 | 83.05 |
| 168.18 | 112.27 | 227.27 | 195.45 |
| 87.52 | 81.40 | 87.52 | 99.54 |
| 63.43 | 112.50 | 134.37 | 115.62 |
| 73.25 | 56.53 | 100.30 | 100.00 |
| 87.17 | 79.48 | 68.20 | 97.43 |
| 89.67 | 99.56 | 105.27 | 102.63 |
| 101.30 | 95.42 | 95.09 | 100.98 |
| 75.25 | 70.16 | 122.37 | 124.40 |
| 100.00 | 68.71 | 67.17 | 84.10 |
| 60.00 | 60.00 | 76.66 | 88.33 |
| 100.00 | 62.55 | 57.23 | 96.17 |
| 94.13 | 87.34 | 105.86 | 100.61 |
| 97.29 | 95.33 | 106.14 | 120.88 |

| **Hyo+1** | **Hyo+2** | **Hyo+3** | **Hyo+4** |
| --- | --- | --- | --- |
| 99.34 | 65.070 | 74.93 | 96.01 |
| 78.02 | 92.560 | 80.38 | 72.58 |
| 84.52 | 64.080 | 60.23 | 91.08 |
| 85.15 | 53.690 | 92.47 | 91.00 |
| 85.32 | 31.740 | 49.51 | 75.78 |
| 54.93 | 41.380 | 94.68 | 90.84 |
| 78.15 | 50.090 | 47.04 | 92.07 |
| 62.93 | 87.880 | 84.56 | 78.09 |
| 88.09 | 71.570 | 95.02 | 95.74 |
| 73.10 | 86.150 | 83.47 | 76.04 |
| 87.12 | 57.010 | 58.37 | 68.04 |
| 75.83 | 87.910 | 74.16 | 91.85 |
| 83.09 | 70.640 | 88.03 | 86.85 |
| 75.20 | 78.140 | 87.03 | 90.12 |
| 62.03 | 49.240 | 83.57 | 75.97 |
| 45.50 | 40.010 | 85.48 | 74.08 |
| 71.81 | 69.670 | 66.07 | 85.04 |
| 32.61 | 91.230 | 71.73 | 92.04 |

| **Vard +1** | **Vard +2** | **Vard +3** | **Vard +4** |
| --- | --- | --- | --- |
| 60.29 | 76.270 | 74.09 | 70.21 |
| 44.68 | 40.310 | 74.37 | 75.31 |
| 74.12 | 44.200 | 81.40 | 92.72 |
| 85.14 | 88.070 | 80.96 | 90.37 |
| 91.01 | 77.340 | 104.29 | 107.42 |
| 50.16 | 45.150 | 76.25 | 98.32 |
| 81.63 | 91.150 | 92.85 | 97.27 |
| 61.37 | 60.770 | 67.66 | 70.05 |
| 93.87 | 83.220 | 108.38 | 106.77 |
| 80.11 | 78.356 | 78.16 | 81.09 |
| 60.26 | 61.050 | 83.42 | 90.26 |
| 61.11 | 55.180 | 77.40 | 81.11 |
| 83.89 | 71.150 | 88.46 | 91.10 |
| 100.26 | 60.100 | 101.90 | 95.21 |
| 88.14 | 69.230 | 57.17 | 55.09 |
| 84.79 | 86.140 | 62.16 | 71.62 |
| 110.58 | 105.090 | 139.21 | 130.19 |
| 91.29 | 96.910 | 64.04 | 73.31 |

| **BAY +1** | **BAY +2** | **BAY +3** | **BAY +4** |
| --- | --- | --- | --- |
| 84.72 | 87.19 | 75.36 | 101.47 |
| 101.49 | 108.97 | 79.80 | 102.24 |
| 51.54 | 127.31 | 103.60 | 106.44 |
| 85.22 | 131.92 | 102.90 | 108.17 |
| 82.68 | 82.08 | 95.52 | 86.56 |
| 85.79 | 88.47 | 91.15 | 96.51 |
| 65.09 | 77.45 | 81.45 | 99.27 |
| 49.73 | 63.15 | 81.57 | 113.42 |
| 76.23 | 73.31 | 94.17 | 81.83 |
| 98.10 | 97.34 | 84.06 | 85.38 |
| 64.15 | 98.70 | 102.59 | 105.19 |
| 80.75 | 85.00 | 100.00 | 89.50 |
| 102.27 | 86.56 | 86.33 | 92.48 |
| 62.50 | 81.25 | 121.87 | 134.37 |
| 80.00 | 78.75 | 99.25 | 130.00 |
| 97.66 | 64.13 | 81.63 | 97.08 |
| 82.38 | 80.82 | 110.10 | 106.47 |
| 83.11 | 81.57 | 88.37 | 99.34 |

| **K+Hyo +1** | **K+Hyo +2** | **K+Hyo +3** | **K+Hyo +4** |
| --- | --- | --- | --- |
| 70.91 | 65.07 | 74.93 | 106.01 |
| 85.04 | 82.56 | 90.38 | 92.58 |
| 94.52 | 94.08 | 80.23 | 101.08 |
| 77.42 | 73.69 | 92.47 | 91.00 |
| 75.32 | 61.74 | 79.51 | 95.78 |
| 87.93 | 81.38 | 94.68 | 90.84 |
| 88.15 | 80.09 | 97.04 | 102.07 |
| 92.93 | 87.88 | 84.56 | 98.09 |
| 78.09 | 61.57 | 95.02 | 100.74 |
| 45.82 | 66.15 | 73.47 | 76.04 |
| 66.28 | 97.01 | 98.37 | 88.04 |
| 50.83 | 47.91 | 74.16 | 71.85 |
| 73.09 | 70.64 | 88.03 | 86.85 |
| 77.52 | 58.14 | 87.03 | 90.12 |
| 82.03 | 79.24 | 63.57 | 75.97 |
| 71.54 | 80.01 | 85.48 | 74.08 |
| 97.81 | 89.67 | 100.07 | 85.04 |
| 89.61 | 81.23 | 91.73 | 92.04 |

| **K+Vard +1** | **K+Vard +2** | **K+Vard +3** | **K+Vard +4** |
| --- | --- | --- | --- |
| 75.05 | 95.09 | 103.62 | 105.97 |
| 100.77 | 85.32 | 78.37 | 92.27 |
| 94.00 | 105.50 | 87.25 | 95.75 |
| 80.31 | 80.08 | 108.27 | 104.02 |
| 65.51 | 70.11 | 65.05 | 102.29 |
| 81.38 | 83.56 | 84.47 | 74.42 |
| 83.57 | 133.09 | 108.21 | 108.93 |
| 48.33 | 65.41 | 59.16 | 109.58 |
| 67.73 | 70.60 | 132.58 | 135.46 |
| 77.15 | 85.23 | 101.23 | 91.21 |
| 94.40 | 103.57 | 70.24 | 85.23 |
| 90.00 | 80.25 | 90.50 | 110.75 |
| 91.41 | 60.08 | 80.47 | 98.28 |
| 78.00 | 90.66 | 74.66 | 97.66 |
| 90.25 | 83.52 | 88.97 | 99.76 |
| 50.49 | 120.79 | 92.40 | 106.27 |
| 85.15 | 75.07 | 107.00 | 91.31 |
| 87.29 | 80.27 | 93.51 | 93.51 |

| **K+BAY +1** | **K+BAY +2** | **K+BAY +3** | **K+BAY +4** |
| --- | --- | --- | --- |
| 82.50 | 99.06 | 127.50 | 83.12 |
| 92.08 | 85.41 | 115.41 | 97.50 |
| 168.00 | 112.28 | 227.14 | 195.42 |
| 101.10 | 95.20 | 95.94 | 101.10 |
| 100.00 | 77.82 | 57.58 | 96.10 |
| 72.19 | 77.00 | 64.97 | 95.45 |
| 75.14 | 96.19 | 122.51 | 123.97 |
| 62.17 | 94.96 | 94.96 | 98.39 |
| 87.50 | 81.25 | 87.50 | 99.56 |
| 94.14 | 87.38 | 105.40 | 100.45 |
| 89.41 | 107.24 | 105.29 | 102.50 |
| 89.41 | 107.24 | 105.29 | 102.50 |
| 60.24 | 81.36 | 76.39 | 88.81 |
| 100.13 | 79.41 | 67.05 | 84.11 |
| 97.41 | 95.22 | 105.96 | 120.87 |
| 63.26 | 112.44 | 134.28 | 115.51 |
| 73.04 | 103.47 | 100.43 | 100.00 |
| 65.36 | 83.17 | 98.82 | 95.69 |
